# Performance Evaluation of Cementitious Composites Incorporating Nano Graphite Platelets as Additive Carbon Material

**DOI:** 10.3390/ma15010290

**Published:** 2021-12-31

**Authors:** Farhan Ahmad, Arshad Jamal, Mudassir Iqbal, Muwaffaq Alqurashi, Meshal Almoshaogeh, Hassan M. Al-Ahmadi, Enas E. Hussein

**Affiliations:** 1 Civil Engineering Department, Faculty of Engineering Sciences, National University of Modern Languages, Rawalpindi 44000, Pakistan; engrfarhan51@gmail.com; 2 Department of Civil and Environmental Engineering, College of Design and Built Environment, King Fahd University of Petroleum & Minerals, Dhahran 31261, Saudi Arabia; ahmadi@kfupm.edu.sa; 3 Interdisciplinary Research Center of Smart Mobility and Logistics (IRC-SML), King Fahd University of Petroleum & Minerals, Dhahran 31261, Saudi Arabia; 4 State Key Laboratory of Ocean Engineering, Shanghai Key Laboratory for Digital Maintenance of Buildings and Infrastructure, School of Naval Architecture, Ocean and Civil Engineering, Shanghai Jiao Tong University, Shanghai 200240, China; mudassiriqbal29@sjtu.edu.cn; 5 Civil Engineering Department, University of Engineering and Technology, Peshawar 25120, Pakistan; 6 Department of Civil Engineering, College of Engineering, Taif University, P.O. Box 11099, Taif 21944, Saudi Arabia; m.gourashi@tu.edu.sa; 7 Department of Civil Engineering, College of Engineering, Qassim University, Buraydah 51452, Saudi Arabia; m.moshaogeh@qu.edu.sa; 8 National Water Research Center, P.O. Box 74, Shubra El-Kheima 13411, Egypt

**Keywords:** nano graphite platelets, concrete, mechanical properties, cementitious composites durability properties

## Abstract

Nano graphite platelets (NGPs) belong to the carbon family and have a huge impact on the construction industry. NGPs are used as multi-functional fillers and have the potential to develop reinforcing within cementitious composites. In this paper, NGPs were incorporated in cementitious composites to investigate the effects of NGPs on the fresh, mechanical, durability, and microstructural properties of concrete. Five mixes were prepared with intrusion of NGPs (0%, 0.5%, 1.5%, 3%, and 5% by weight of cement). The properties studied involved workability, air content, hardened density, compressive strength, tensile strength, flexural strength, sorptivity, ultrasonic pulse velocity (UPV), water absorption, and external sulfate attack. The workability and percent air content decrease by 22.5% and 33.8%, respectively, for concrete with 5% NGPs compared to the control mix. The specimens containing 5% of NGPs revealed the hardened density, compressive, tensile, and flexural strength to increase by 11.4%, 38.5%, 31.6%, and 44.34%, respectively, compared to the control mix. The results revealed that the incorporation of 5%NGPs in cementitious composites reduces the sorptivity and water absorption by 32.2% and 73.9%, respectively, whereas, it increases the UPV value by 7.5% compared to the control mix. Furthermore, the incorporation of NGPs provided better resistance against external sulfate attacks. SEM–EDX spectroscopy was carried out to investigate its microstructural analysis.

## 1. Introduction

Nanotechnology and its application in the construction industry have revolutionized abilities, visions, and expectations, e.g., by controlling the material world, attracting investigators to inspect their peculiar characteristics, with regard to reinforcing within the cementitious matrix. Advancements in nanotechnology play a huge role in the construction industry by incorporating new nanomaterials or fibers in concrete, which plays a tremendous role in enhancing its mechanical properties. Today, concrete is more durable and stronger than in the past, because of better knowledge and skills at the nano level, and the improved mechanical characteristics of concrete. These nanomaterials exhibit extraordinary strength in cementitious composites by reinforcing nano levels. Moreover, cement is being replaced by several additives to enhance the cementitious performance of concrete and eco-friendly–sustainable construction [1,2,3]. These nanomaterials have created a new generation of cementitious composites, having excellent resistance against crack propagation at the nano level, with outstanding energy absorption capabilities before failure. For instance, several nanomaterials, such as nano graphite platelets [4,5,6], graphene oxide [7], carbon nanotubes [8], carbon nano fibers [9], graphene [10], nano-silica [11], nano titanium dioxide (TiO_2_) [12], and nano clay have been utilized to date to reinforce the cementitious composites (cement paste, mortar, and concrete).

Graphene oxide (GO), nano graphite platelets (NGPs), graphene-based derived, carbon nanotubes (CNTs), etc., are carbon-based nanomaterials that might definitely result in “smart” properties [5,13]. Chougan, M., E. Marotta, F. R. Lamastra, F. Vivio, G. Montesperelli, U. Ianniruberto, S. H. Ghaffar, M. J. Al-kheetan, and A. Bianco [14] examined the behavior of density, compressive strength, flexural strength, microstructure, and permeability properties of concrete with different commercial nano graphite content (i.e., 0.01%, 0.1%, and 0.2%), and noted a substantial increase in density and mechanical characteristics—up to 16% and 30%, respectively. Moreover, a substantial decline in permeability was reported. Meng, W. and K. H. Khayat [4] studied the influence of two different nanomaterials, GNPs and CNFs, on the mechanical behavior of UHPC. The nanomaterial content ranged from 0 to 0.3%; the authors reported an increment from 5 to 8 MPa in compressive strength. They also reported that 56%, 59%, and 276% enhancement was examined in tensile strength, flexural strength, and toughness, respectively. Ahmad, F. [5] studied the effect of NGPs on the mechanical and durability performance of plastic concrete. The contents of NGPs (i.e., ranging from 0 to 5%) were incorporated, and the authors found that the compressive, tensile, and flexural strength of plastic concrete containing 5% NGPs increased by 13.5%, 15.5%, and 31.4%, respectively, in comparison to plastic concrete without nanoparticles. Moreover, an increase in the UPV values and a decrease in the sorptivity values were observed when NGPs were incorporated. Giannakopoulou, P. P., A. Rogkala, P. Lampropoulou, M. Kalpogiannaki, and P. Petrounias [6] studied the influence of nano MgO and fly ash (industrial byproduct) on the cement performance and reported that the influence of nano MgO on the physicomechanical performance of cement was more critical compared to fly ash. Moreover, it was reported that the samples containing 3–4% of MgO yield a more suitable performance on the physicomechanical behavior of cement. Cui, X., S. Sun, B. Han [15] examined the effect of NGPs on the thermal, mechanical, and electromagnetic properties of composites and observed that an enhancement, up to 1.5 fold in hardness, 71% reduction in abrasive loss, 77% increase in thermal conductivity, and a 73% decrease in abrasive depth was reported at 5% NGPs, in comparison to cementitious composites without nanomaterials. Yu, L. and R. Wu [16] used the fine recycled aggregate and studied its performance by introducing graphene oxide (GO) in UHPC. They described that comparable results in mechanical characteristics of UHPC, containing recycled fine aggregate, were obtained, when compared to concrete with natural sand. A significant increase in strength characteristics, such as compressive, tensile, and flexural strength, was reported, i.e., up to 197%, 160%, and 184%, respectively, with the incorporation of 0.02% GO in cementitious composites [17]. Sharma, S. and N. Kothiyal [7] investigated that, with the incorporation of 1% GO (by mass of cement) in concrete composites, a decrement in porosity from 25.21% to 10.61% was observed.

NGPs are carbon-based nanomaterials derived from graphite. NGPs have a two-dimensional (2D) structure consisting of several layers of graphene and the diameter of particles ranging from submicron up to 100 μm [18,19]. NGPs, being nanofillers, significantly reduce the porosity and reinforce the cementitious composites in microstructure, thereby improving their density and hardness significantly [20,21]. Because of larger surface areas and better surface structures, NGPs provide excellent interaction among the surrounding hydration products, which not only help in stopping the crack generation, but they also, due to the 2D plate-like structure, divert the crack path, thus help in delaying crack propagation [22,23]. Liu, Q., Q. Xu, Q. Yu, R. Gao, and T. Tong [24] examined the influence of GNPs on cement mortar and reported that the compressive strength was improved by 36%. Kim, J.-S., J.-Y. Lee, Y.-H. Kim, D. Kim, J. Kim, and J.-G. Han [3] investigated the influence of feldspar on the compressive strength of sand-based mortar by incorporating feldspar (i.e., 5% to 10%) as a partial substitution of fine aggregate. It was examined that the compressive strength of the feldspar-derived mortar was about 1.1–4.5 times higher than normal sand mortar. Moreover, it was reported that feldspar-based mortar can be used as eco-friendly construction material and is expected to reduce cement dependency attributed to the reinforcement property of feldspar. Liu, L., G. Yang, J. He, H. Liu, J. Gong, H. Yang, W. Yang, and P. Joyklad [2] examined the effect of blended fibers on the mechanical characteristics of cement mortar at high temperatures (i.e., 20 °C to 750 °C) and revealed that the cementitious composites containing blended fibers performed better under fire. Moreover, it was reported that the incorporation of calcite powder to fiber-reinforced cementitious composites increased the mechanical strength, across the board, at all temperatures. Yang, M., G. Chen, N. Cao, Y. Zhang, and Y. Wang [25] studied the influence of GNP on durability properties, and compressive and flexural strength of cement mortar, by incorporating GNP, ranging from 0.2% to 0.6% by weight of cement. It was revealed that an approximate 10% and 8% increase in compressive and flexural strength, respectively, were observed at 0.2 wt% of GNP. Moreover, it was reported that significant improvement in acid resistance and durability of cement mortar was observed. Akbar, A., K. Liew, F. Farooq, and R. A. Khushnood [20] investigated the combined effect of MWCNTs and GNMPs on the behavior of cementitious composites and reported that the mechanical characteristics were significantly enhanced. Moreover, it was found that the addition of GNMPs provided excellent resistance against a sulfate attack and water absorption. Mohammed, A., J. Sanjayan, W. Duan, and A. Nazari [26] reported that incorporating graphene oxide (GO) in cementitious composites significantly improves the impermeability and corrosion resistance owing to its nanofiller effect and pore refinement agent. It was reported that by the addition of graphite platelets, water permeability, chloride diffusion, and chloride migration in concrete could be abridged [27,28].

A brief assessment of the current literature shows that, to date, there are few published studies concerning the role NGPs on the behavior of concrete, suggesting that a [7,15,25,29] comprehensive study associated with the performance of NGPs in cementitious composites is missing. This investigation was conducted to develop nano-reinforced concrete. The authors considered the NGP dosage limited to 5% in the viewpoint of the previous literature. Khushnood, R. A. and A. Nawaz [29] looked at ordinary concrete composite by adding different percentages of NGP by weight of the cement. This study explores fresh properties (i.e., workability and air content) and hardened properties, in terms of mechanical, durability, and microstructural properties of concrete, by adding different contents of NGPs (i.e., 0.5%, 1.5%, 3%, and 5%) by weight of the cement. We examined the effect of 0–1% (by mass) of GNMPs on the freeze–thaw resistance of concrete. Cui, X., S. Sun, B. Han, X. Yu, J. Ouyang, S. Zeng, and J. Ou [15] examined the effect of 5% NGPs on strength, thermal, and electromagnetic properties of cementitious composites. Mechanical properties studied include hardened density, compressive, tensile, and flexural strength whereas, durability properties include sorptivity, UPV, water absorption, and external sulfate attack. A dispersion test of NGPs was carried out to properly disperse NGPs in concrete composites, and the agglomeration and staking behavior of NGP particles were explained. Moreover, the reinforcing mechanism of the NGPs modified cementitious composites, the interaction among NGPs and cement hydration products were explained by using SEM–EDX spectroscopy.

The remainder of this paper is structured as follows. Section 2 provides a detailed description of different materials used for this research, such as cement and modifier types, dispersion scheme of NGPs, concrete mix proportioning, and specimen preparation. Section 3 presents a comprehensive overview of the adopted experimental program. Section 4 presents the study results and intuitive discussion in light of similar previous studies. Finally, Section 5 summarizes the key findings and a brief outlook for future studies.

## 2. Materials

OPC (Type-1) obtained from BESTWAY cement factory was utilized for concrete production as per ASTM C-150 standard. Table 1 summarizes the general properties of OPC. Table 1 shows the oxide composition of cement, which was obtained experimentally through the X-ray fluorescence (XRF) technique.

Locally available “Lawrencepur sand” was utilized as a fine aggregate, having a fineness modulus of 2.25 as per ASTM C-33 standard. Coarse aggregate utilized in this research work was obtained from the Taxila brand (Margalla), Pakistan, having a maximum particle size of 20 mm, and superplasticizer (polycarboxylate based) was obtained from BASF. Ordinary tap water was used for both castings and curing, having a PH range between 6.5 and 7. The general characteristics of aggregates are enlisted in Table 2.

Nano graphite platelets (NGPs) were commercially procured in powdered form. To investigate the structural characterization of commercial NGPs, SEM, EDX, XRD, and XRF analyses were carried out. SEM, EDX, XRF, and XRD patterns of NGPs are discussed in Section 4.1. NGPs tend to agglomerates and rebind to each other via van der Waals forces. For effective dispersion of NGPs, a natural surfactant, called acacia gum, was used. Acacia gum (AG) was found to be very effective for this purpose [30,31]. Table 3 shows the elemental composition of acacia gum obtained through EDX spectra.

### 2.1. Dispersion Scheme of NGPs

**Figure 1:**
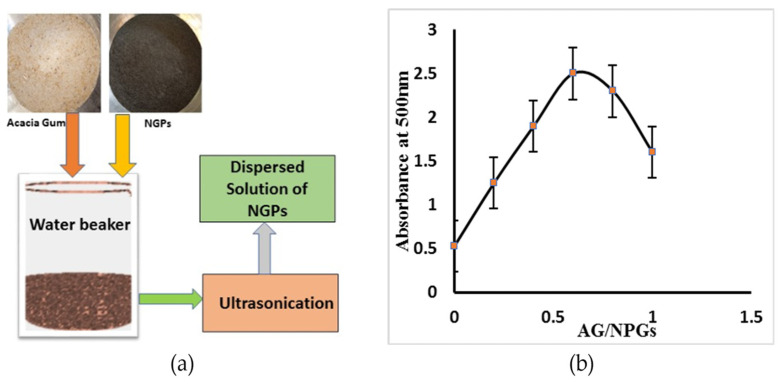
(**a**) Flow chart of NGP dispersion; (**b**) UV–Vis spectroscopy analysis results.

NGPs are carbon-based nanomaterials and have the tendency to agglomerate and stack to one another, owing to the strong van der Waals forces among the nanoparticles. Moreover, due to their hydrophobic nature, it doesn’t interact with water-based systems [20,31]. Due to the large surface area of NGPs, the van der Waals forces exist among nanoparticles that resist its dispersion in cementitious composites. To utilize the NGPs effectively, it is essential to equally disperse these particles in concrete composites. For uniform dispersion of NGPs, usually the use of chemical or natural surfactants with mechanical sonication are required [4,29]. In this study, a natural surfactant, i.e., AG was added. AG not only homogenously disperses NGPs, but also increases the retention time, thus helping in the stabilization of dispersed aqueous solution. AG weakens the van der Waals forces by adsorbing on the surface of graphitic particles; hence, helping in the uniform dispersion of NGPs [29,31]. Hence, to obtain a well-dispersed aqueous solution, acacia gum was chosen as a surfactant. The detailed procedure for the dispersion of NGPs is discussed in the following manner; primarily, NGP to AG ratios were chosen, as shown in Table 4 in the current study. In this study, the surfactant–ultrasonication method was adopted to obtain an aqueous dispersed solution. Afterward, the dispersed aqueous solution was further diluted and mixed with the amount of water required for the preparation of concrete. To analyze the dispersion, the sample was taken from the dispersed solution (well diluted) and was tested using UV–Vis spectroscopy to check how effectively NGPs dispersed. The absorbance of the sample solution is usually determined at 500 nm [32]. For each ratio shown in Table 4, the absorbance of a sample solution was checked, and a graph was finally plotted between the absorbance and surfactant (AG)/NGPs ratios. The results reveal that the surfactant/NGP ratio, i.e., 0.6:1, shows maximum absorption, and is considered the optimum dosage for effective dispersion aided with 45 min of sonication, mechanically, as given in Figure 1b. Whereas, Figure 1a shows the complete flow chart of the dispersion phenomena.

### 2.2. Concrete Mix Proportions

In this study, a total of five mixes were prepared, and the mix ratio used was 1:1.86:2.89. The water/cement ratio was kept at 0.45. The cement, sand, and coarse aggregate content were kept constant at 384 kg/m^3^, 715 kg/m^3^, and 1113 kg/m^3^, respectively. Different dosages of NGPs i.e., 0.5%, 1.5%, 3%, and 5% by weight of the cement were added. Table 5 displays the detailed mix proportions. For the preparation of concrete mixes, a tilting drum was used revolving at a speed of 35 rev/min. Five concrete mixes were prepared with one control mix, and the other of NGP incorporated concrete. The concrete mixing was performed in three stages. Initially, the dispersed aqueous solution of NGPs and AG was prepared by using an ultra sonicator. In the second stage, the fine and coarse aggregate, along with 75% of water (dispersed and diluted NGPs) was added and mixed for 4 min. In the last stage, cement, along with 25% of water, was added and mixed for the next 4 min. To achieve workable concrete, a superplasticizer was mixed with water containing dispersed aqueous solution by stirring. After that, the fresh concrete was then kept into the molds, which were properly oiled to obtain the samples of the desired shapes and sizes. After 24 h, the specimens were unmolded and kept in the curing tank up to the age of testing.

### 2.3. Specimen Preparation

To study compressive and tensile properties of concrete, 30 cylindrical specimens of 150 × 300 mm were cast. Whereas, for the analysis of flexural behavior of concrete, 15 prismatic specimens of 100 × 100 × 400 mm^3^ were prepared. For the sorptivity test, cylindrical samples of 50 mm in thickness and 100 mm diameter were prepared. To determine the influence of external sulfate attack on concrete performance, cylindrical specimens of 150 × 300 mm were cast and cured for 28 days, the specimens were then submerged in sodium sulfate solution of 30 g/L for 28 days, and then the specimens before and after the submersion were tested.

## 3. Experimental Methods

**Figure 2:**
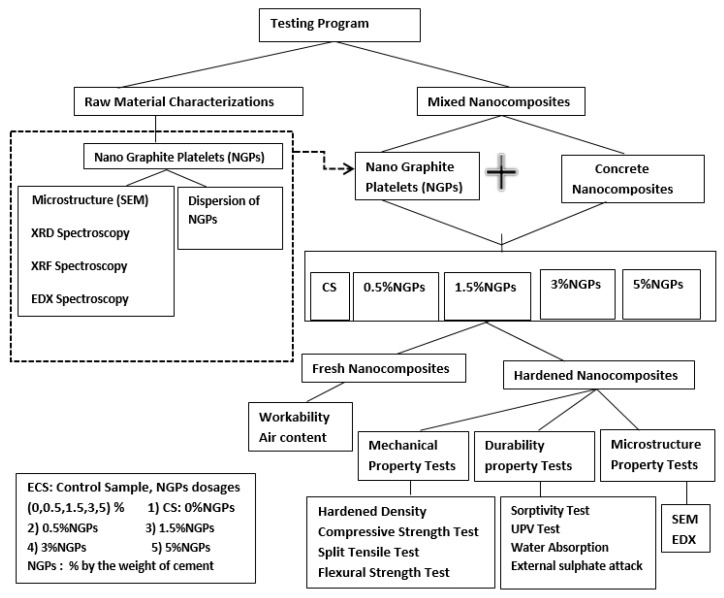
Experimental program flow chart. Reproduced with permission from Ref. [5]. Copyright (2021), Elsevier Ltd.

The comprehensive flow chart of the experimental program is indicated in Figure 2.

### 3.1. Fresh Concrete Properties

The fresh concrete properties include workability and air content. The air content of fresh concrete was evaluated by the pressure method, as per ASTM C231/C231M-17a [33]. Workability was examined by performing a slump test, as per standard ASTM C143 [34]. A slump cone (200 mm lower diameter, 100 mm upper diameter, and 300 mm height) was used to determine slump value, in which the fresh concrete was poured in three layers, and each layer was given 25 blows with a tamping rod. For each slump value, three tests were carried out, and the mean result was considered. The slump values were recorded for different concrete mixes to analyze the effect of NGPs on the fluidity of the nano-reinforced concrete composites.

### 3.2. Hardened Concrete Properties/Mechanical Properties

The hardened concrete properties include hardened density, compressive, flexural, and tensile strength of concrete. The hardened density of concrete specimens was calculated using analytical balance, by evaluating its size and weight at saturated surface dry conditions (SSD) after 28 days. As per ASTM C496/C496M-17 and ASTM C39/C39M-12 [35,36] standards, the compressive and tensile strength of concrete was determined. Cylindrical specimens (150 × 300 mm) were used to find out the compressive and tensile behavior of concrete after a curing period of 28 days. For the determination of compressive and tensile strength, a universal testing machine (UTM) with a load capacity of 1000 kN was used. The compressive strengths of cylindrical concrete specimens were simply determined by dividing the maximum applied load obtained from UTM by the surface area of cylinder. Whereas, the split tensile strength of specimens was determined using the following formula;

(1) $$f_{t}=\frac{2P}{\pi DL}$$

In Equation (1), $f_{t}$ represents the split tensile strength, P denotes the maximum applied load, and D and L denote the diameter and length/height of the concrete cylinder, respectively.

Flexural strength of NGP-reinforced concrete was determined as per ASTM C78/C78M-18 [37], by carrying out a three-point bend test. For each formulation of concrete mix, three specimens were tested at 28 days. A total of 15 prismatic specimens of 100 × 100 × 400 mm^3^ were prepared, and by using the flexural testing machine, the specimens were tested. The flexural strength of the rectangular specimens (i.e., small beams) was determined using the following formula:

(2) $$\sigma =\frac{3Pa}{bd^{2}}$$

In Equation (2), σ represents flexural strength, P is the maximum applied load on a sample, d and b are the thickness and width of the sample, while a is the distance between the nearest support and the line of rupture.

### 3.3. Durability Properties

The durability characteristic of NGP-reinforced concrete were determined based on sorptivity co-efficient, UPV test, water absorption, and external sulfate attack, UPV test. To evaluate the sorptivity co-efficient, the cylindrical specimen 100 × 200 mm was cut into 100 × 50 mm discs, and were tested, as per ASTM C1585-04 [38]. To prevent the side water ingress, both the top surface and circumferential area were covered with epoxy paint and sealed with polyethylene sheets to block the evaporation. Only that surface of specimen is unsealed, which is subjected to water ingress. Through capillary action, the rise of water takes place and is evaluated due to an increase in the weight of the sample. The sorptivity (S) was evaluated using Equation (3).

(3) $$S=I/t^{1/2}$$

In Equation (3), *S* denotes sorptivity coefficient evaluated in mm/min^1/2^, *t* denotes time in minutes, and I=ΔW/Ad, $\Delta W=W_{2}-W_{1}$, *W*_1_ is the dry weight of the sample in grams, *W*_2_ is the sample weight in grams after 4 h of ingression via capillary rise, *A* is the surface area of the exposed surface, and *d* is the water density.

The UPV test was performed to analyze the uniformity and quality of cementitious composites. The cylindrical specimens (150 mm in diameter × 300 mm in length) were tested to determine the UPV value. The UPV values were evaluated as per ASTM C597-09 standard. The two transducers, i.e., transmitter and receiver, were provided at both ends of the cylindrical specimen, and then, the pulse velocity, having a frequency range of 55 kHz, was allowed to travel between the two transducers. The travel time between the two transducers was noted, and the UPV value was obtained by dividing the length of the cylinder by the corresponding time of travel.

Water absorption of NGP-reinforced cementitious composites was determined as per the ASTM C642 standard [39]. For concrete samples at 28-day curing periods, water absorption was calculated using Equation (4) [40]. W1 represents the weight of samples after 24 h of casting by using a highly accurate weighting balance, whereas W2 represents the weight of the saturated surface samples after 28 days of curing. The samples were taken out from the water after 28 days, and their surfaces were towel-dried.

(4) $$\mathrm{Water}\;\mathrm{absorption}\;\left(\%\right)=\left[\frac{W2-W1}{W1}\right]\times 100$$

An external sulfate attack test was conducted to check the resistance of concrete against sulfate. A sulfate attack adversely affects the durability characteristics of concrete. To determine the influence of an external sulfate attack on the concrete performance, cylindrical specimens (150 mm in diameter × 300 mm in length) were cast and cured for 28 days, and then the specimens were submerged in a sodium sulfate solution of 30 g/L for 28 days. The concrete specimens were tested before and after the submersion, and the loss/reduction in compressive strength was noted.

### 3.4. Microstructure Investigation

Study of the microstructure of NGP-reinforced cementitious composites were carried out using SEM and EDX analyses. SEM was carried out according to guidelines mentioned in ASTM C1723 [41]. The SEM analysis was carried out in order to analyze morphological, compositional, or topographical variations in the tested samples. The broken specimens, which underwent a compression test, were utilized for the SEM analysis. To freeze the microstructural features of the sample, the sample was completely dried to stop its hydration process at 28 days. At 110 °C, the specimens were oven-dried for 3 days and then finally coated with a gold layer. Whereas the EDX analysis was performed to examine the elemental percentage composition of the sample. In this, the X-rays were generated as a result of the interaction between the projected electron beam and the proposed sample, which were then detected, and could be used as essential tools for further investigation.

## 4. Results and Discussion

### 4.1. Structural Characterization of NGPs

**Figure 3:**
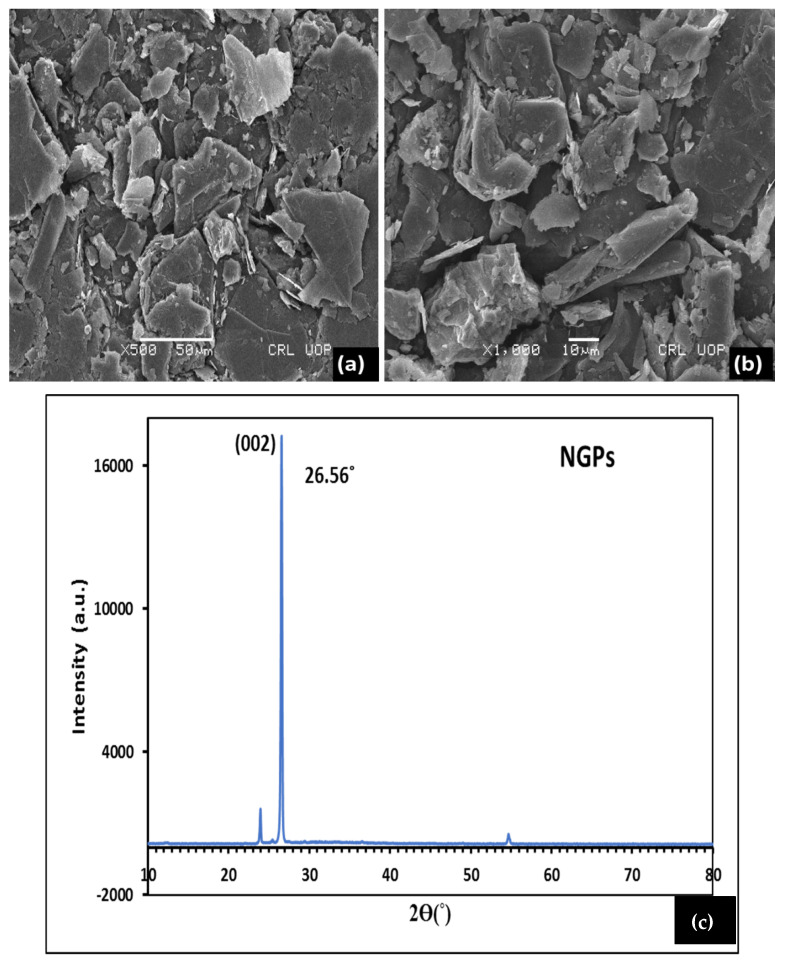
(**a**,**b**) SEM micrographs; (**c**) XRD graph. Reproduced with permission from Ref. [5]. Copyright (2021), Elsevier Ltd.

The analysis of commercially available NGPs were conducted by means of SEM, XRD, XRF, and EDX spectroscopy. SEM, EDX, and XRF spectroscopy were carried out to analyze the morphology/surface texture, chemical composition, and mineral oxide composition of NGPs, respectively. The SEM micrograph shown in Figure 3a,b, indicates that NGP particles exhibit irregular shapes and rough textures. Table 6 shows the elemental composition of NGPs obtained experimentally via the EDX analysis. The results reported in Table 6 reveal the presence of high carbon content, of about 84%, indicating it to be NGPs. The compositions of the oxides of NGPs obtained via the XRF technique are shown in Table 7. The XRD investigation was performed to examine and extract the crystal structure and phase composition of the samples. Figure 3c shows the XRD spectra of the NGPs with the appearance of a diffraction peak at 2θ = 26.56°, which is allocated to the (002) diffraction peak of NGPs. The thickness of NGPs, the interlayer distance, and the number of graphene layers evaluated from XRD data are 40.0 nm, 0.335 nm, and 115, respectively. The average crystallite size obtained from the three peaks was 45.7 nm.

### 4.2. Fresh Concrete Properties

#### 4.2.1. Workability

**Figure 4:**
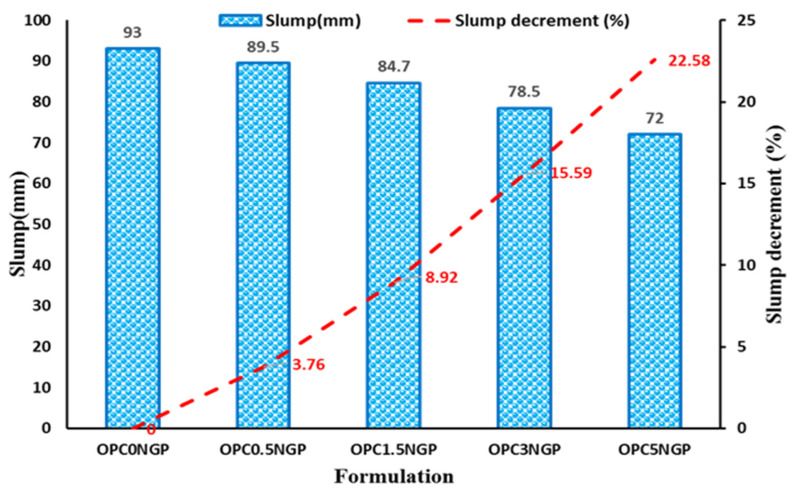
Effect of NGPs on the workability of concrete.

The workability of nano-reinforced concrete was examined using a slump test. Figure 4 shows the results of the slump test for different concrete mixes. The mix, OPC5NGP with 5% NGPs were observed to have a significantly lower slump value followed by OPC3NGP, OPC1.5NGP, and OPC0.5NGP, in comparison with control mix OPC0NGP with 0% NGPs. The results of the NGP-reinforced concrete composites indicate that the slump value declines with an increase in the percentage of NGP content. Therefore, the incorporation of NGPs in concrete composites reduces workability. The effect of NGPs on the workability of concrete, i.e., decrease in fluidity and increase in viscosity of concrete composites, is consistent with the results of previously published works [5,42,43]. The very fine particle size and high surface area of nanomaterials adsorb water from the fresh mix to wet their nano sheets [44,45,46]. Thus, with the increase in NGP content in the concrete mixes, the slump value declines linearly.

#### 4.2.2. Air Content

**Figure 5:**
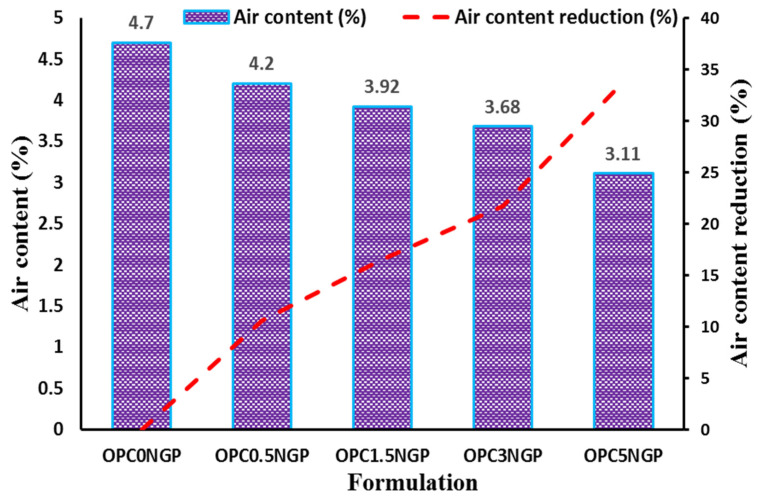
Influence of NGPs on air content (%) of fresh concrete and percentage reduction in air content.

Air content of NGP-incorporated fresh concrete was obtained by the pressure method, as per the ASTM C231/C231M-17a [33] standard using a pressure air meter. Air content is directly related to the density of cementitious composites at the initial stage. The higher the percentage of air content, the lower the density of cementitious composites [20,47]. This relation between air content and density can be justified as material containing larger pore sizes, and more porosity will have higher air content; hence, fresh density will be lower. The presence of the higher air content in concrete increases the workability of concrete, but declines the density and strength of concrete [48]. The influence of NGPs on the air content of concrete and the percentage reduction of air content concerning different dosages of NGPs are reported in Figure 5. The results show that with the increase in dosages of NGPs, the air content reduced. The mix, OPC5NGP, was examined to have significantly lower air content, tailed by OPC3NGP, OPC1.5NGP, and OPC0.5NGP in comparison with control mix OPC0NGP, with 0% NGPs. The mix containing 5% NGPs indicated a maximum reduction in air content of about 33.83% compared to the control mix, as reported in Figure 5. This significant reduction in air content is because of the incorporation of nanoparticles, which enhance the fresh density of concrete, thereby reducing the percent air content [5]. Similar results were reported, with a decrease in air content, an increase in fresh density, and an increase in NGPs content [20]. Uniformly and homogenously dispersed NGPs not only decrease the air content, but also increase the density of intruded concrete, resulting in a dense microstructure due to the pore refining and filler effect of nano intrusions.

### 4.3. Hardened Concrete Mechanical Properties

#### 4.3.1. Hardened Density

**Figure 6:**
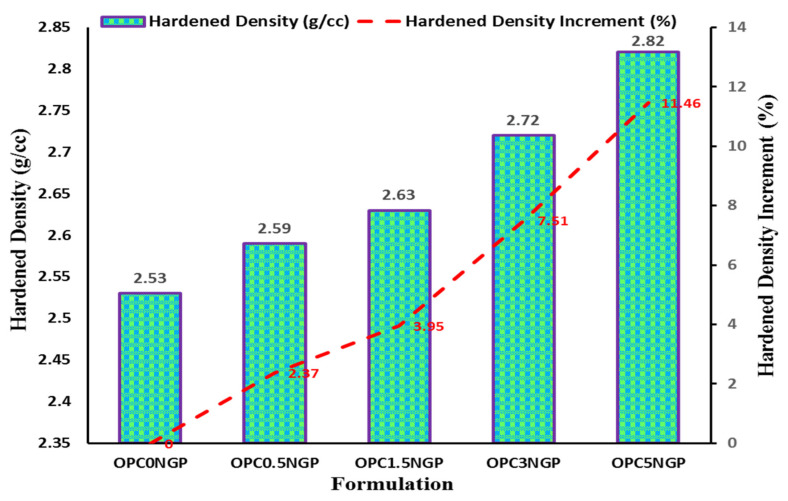
Hardened density (g/cc) and percentage increase in hardened density.

The evolution of density for samples containing different percentage contents of NGPs (i.e., 0.5%, 1.5%, 3, and 5% by mass of cement) at 28 days is shown in Figure 6. The existence of the NGPs induced a density enhancement in concrete in comparison with the concrete containing 0% NGPs. The results reveal that nano-reinforced concrete containing different contents of NGPs (i.e., 0.5%, 1.5%, 3%, and 5%) have approximately 2.37%, 3.95%, 7.5%, and 11.46% enhancement in density, with reference to a control mix without NGPs, as shown in Figure 6. The mix containing 5% NGPs displays a maximum enhancement in density of about 11.46%, referenced to the control mix. This increase in density has to be related to the filler effects of nano particles, which not only reduce the porosity by filling small holes at nano level, but also provide excellent surface interaction with the host matrix, resulting in dense microstructure [20,49]. According to several authors, the incorporation of nanofillers (GO, nG, GNPs, GONPs, etc.) in cementitious composites significantly increase their hardness and density [14,21,50,51]. This improvement is credited to its capability as a pore refining agent, thus causing reduction of porosity and enhanced densification of cementitious composites. Chougan, M., E. Marotta, F. R. Lamastra, F. Vivio, G. Montesperelli, U. Ianniruberto, S. H. Ghaffar, M. J. Al-kheetan, and A. Bianco [14] noted an approximate 15% enhancement in density by introducing nano graphite (nG) in cementitious composites.

#### 4.3.2. Compressive Strength

**Figure 7:**
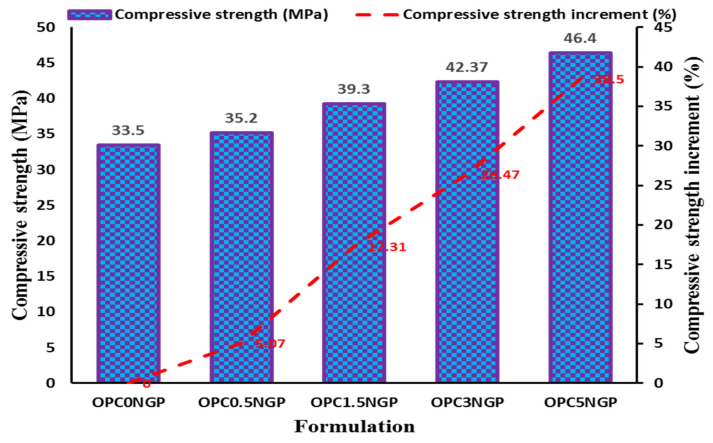
Effect of NGPs on compressive strength of concrete.

Compressive strength is an essential property of concrete that measures its ability to carry loads and used in the reinforced concrete design. In the present study, the compressive strength of nano-reinforced concrete, having different dosages of NGPs, was examined, as per the ASTM C39 [36] standard. The compressive strength was evaluated with the help of a universal testing machine (UTM). Figure 7 shows the compressive strength results and the percentage variation in compressive strength of different nano-reinforced concrete composites at a 28-day curing period. The results reported that concrete mixes containing different contents of NGPs have better compressive strength compared to the control mix, OPC0NGP. The mix containing 5% NGPs, OPC5NGP, has the maximum enhancement, followed by OPC3NGP, OPC1.5NGP, and OPC0.5NGP. It can be seen that, with an increase in the percentages of NGPs in the concrete composites, the compressive strength gradually increases. The maximum enhancement in compressive strength of about 38.5% was observed for a concrete mix containing 5% NGP in comparison with the control mix. Whereas, a minimum increase in strength of 5.07% was observed for the mix with 0.5% NGPs. Past works have reported that incorporation of a small number of nanomaterials (NGPs, nG, GO, GNMP, etc.) in cementitious composites significantly enhanced the compressive strength at a large scale [14,31,52,53]. Chougan, M., E. Marotta, F. R. Lamastra, F. Vivio, G. Montesperelli, U. Ianniruberto, S. H. Ghaffar, M. J. Al-kheetan, and A. Bianco [14] reported that an approximate 30% increase in compressive strength was found by incorporating a small amount of nano-graphite (0.2% of nG by mass of cement). NGPs are the nanofillers that significantly enhance the density of concrete composites owing to the decrease in porosity and strengthening of concrete composites in the microstructure. Moreover, the increase in compressive strength may be credited to the strong interfacial cohesion/bonding among the cementitious matrix and the nanofillers, which cause the densification of cementitious composites due to its nanofiller effect [5,14,53]. The increase in strength of NGP-reinforced concrete is because of the incorporation of nanomaterials that reinforced the concrete composites at the nano level, thus producing an enhancement in the strength compared to the control mix.

#### 4.3.3. Split Tensile Strength

**Figure 8:**
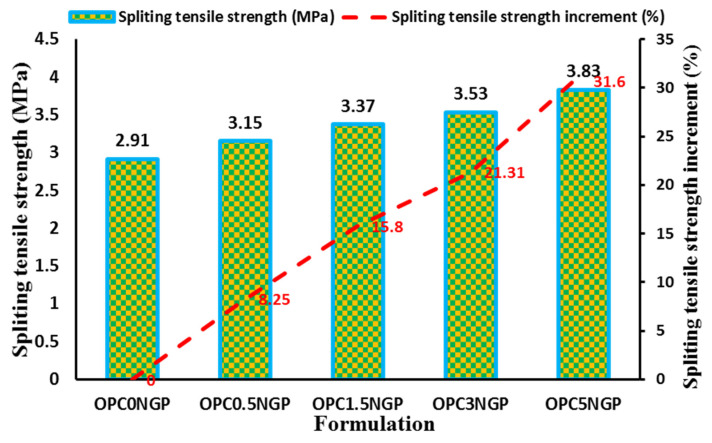
Effect of different contents of NGPs on split tensile strength of concrete.

The splitting tensile strength test is widely used to examine a concrete specimen’s resistance to elongation. This test is performed on a cylindrical specimen in accordance with ASTM C496/C496M-17 [35]. The UTM was used to measure the tensile strength of nano-reinforced concrete specimens. Figure 8 shows the effect of different contents of NGPs on the tensile strength of concrete composites and the percentage increase in tensile strength, respectively. The results show that concrete mixes containing different contents of NGPs have better tensile strength in comparison with the control mix, OPC0NGP. The mix with 5% NGPs, OPC5NGP has the maximum enhancement followed by OPC3NGP, OPC1.5NGP, and OPC0.5NGP. We should note that the concrete composites containing 5% NGPs indicate maximum enhancement in tensile strength of 31.6%, and a minimum improvement of 8.25% was observed for a mix with 0.5% NGPs, by a mass of cement at 28 days of curing. Several investigations on the impact of NGP intrusion in cementitious composites were carried out and examined, showing that NGPs accelerate hydration, increase hardness and density, enhance tensile and flexural strength, and develop ultra-strong interfacial bonding and interlocking between the cementitious matrix and the nanofillers [14,42,52]. Due to the nanofiller effect and high surface area of NGPs, the reduction in porosity and densification of the microstructure at the nano level occurs, producing an enhanced mechanical strength. Thus, the increase in tensile strength of NGP-reinforced concrete is because of the incorporation of NGPs, which strengthened the concrete composites at the nano level [5]. The reinforcing effect of NGPs on the strength of cementitious composites makes NGPs promising reinforcement agents.

#### 4.3.4. Flexural Strength

**Figure 9:**
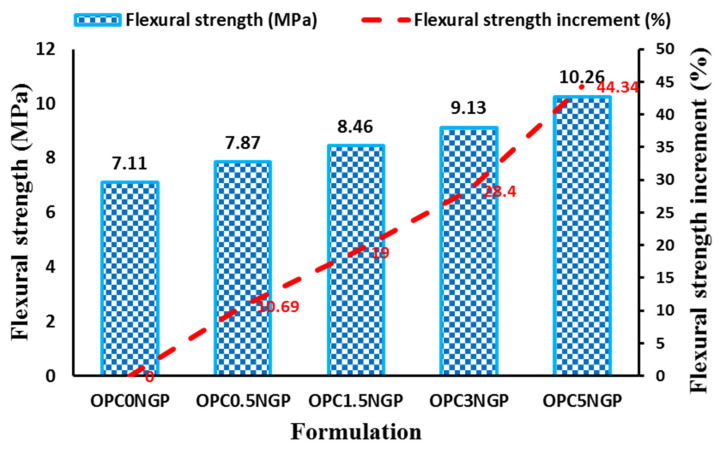
Influence of NGPs on flexural strength of concrete and percentage increase in flexural strength.

The flexural strength test determines the ability of concrete to counter bending loads and was performed as per the ASTM C78/C78M-18 [37] standard. The flexural strength of nano-reinforced concrete composites was determined using the three-point bend test by a flexural testing machine. Figure 9 shows the flexural strength test results of nano-reinforced concrete containing different contents of NGPs. It can be examined that the flexural strength gradually increases with an increase in dosages of NGPs. The results show that nano-reinforced concrete, with different dosages of NGPs (i.e., 0.5%, 1.5%, 3%, and 5%), have approximately 10.69%, 19%, 28.4%, and 44.34% enhancement in flexural strength, with reference to a control mix without nano particles, as indicated in Figure 9. The maximum improvement in flexural strength of about 44.34% was observed for a concrete mix containing 5% NGPs in comparison with the control mix. Whereas, a minimum increase in flexural strength of 10.69% was examined for the mix with 0.5% NGPs. The effect of NGPs on flexural strength is well reflected in previously published studies [4,20,52,54]. The increase in flexural strength of nano-reinforced concrete may be credited to the filler effect of NGPs, which strengthened the composites at a nano level, thereby enhancing the hardness and density of concrete composites [14,50]. Furthermore, the increment in flexural strength of concrete may be due to the very fine particle size, high surface area, and crack divergence capability of NGPs [20,55].

### 4.4. Durability Properties

#### 4.4.1. Sorptivity

**Figure 10:**
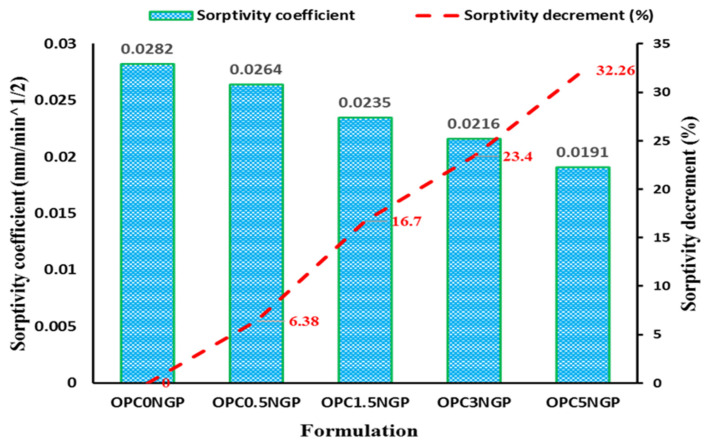
Effect of NGPs on sorptivity of concrete and percentage decrement in sorptivity values.

Sorptivity measures the capacity of porous media to absorb water through capillary actions. The sorptivity coefficient of concrete provides a good measure of the durability of concrete, as many chemicals can penetrate the microstructure of concrete from soils and water by capillary actions. Materials with a high sorptivity coefficient are more susceptible to degradation, which reduces their toughness. The sorptivity coefficient is evaluated by determining the increase in weight of the sample caused by the water absorption (only through one surface of the specimen). The amount of water absorbed by the samples having different dosages of NGPs (i.e., 0.5%, 1.5%, 3%, and 5%) through capillary rise per unit area is shown in Figure 10. The results reported that the sorptivity (mm/min^1/2^) for the concrete mixes containing different content of NGP inclusion has a lesser value than the control mixed. It was observed that the percentage decrease in sorptivity was maximum for a mix containing 5% NGPs, which was reported to be 32.3%, and the minimum reduction was noted to be 6.4% of mix prepared with 0.5% NGPs, compared to the control mix. Ahmad, F. [5] studied the effect of several contents of NGPs (i.e., ranging from 1 to 5% by mass of cement) on cementitious composites, and found that maximum reduction in the sorptivity value occurred when 5% of NGPs was incorporated. Mohammed et al. reported that the incorporation of graphene oxide (GO) affects the transport properties in the cement matrix, which strengthens the microstructure and improves the gel pores of the matrix [42,53,56]. The existence of NGPs decreases the porosity and reinforces the composites at the nano level, resulting in enhanced densification, and reducing the associated water absorption [57].

#### 4.4.2. Ultrasonic Pulse Velocity (UPV)

**Figure 11:**
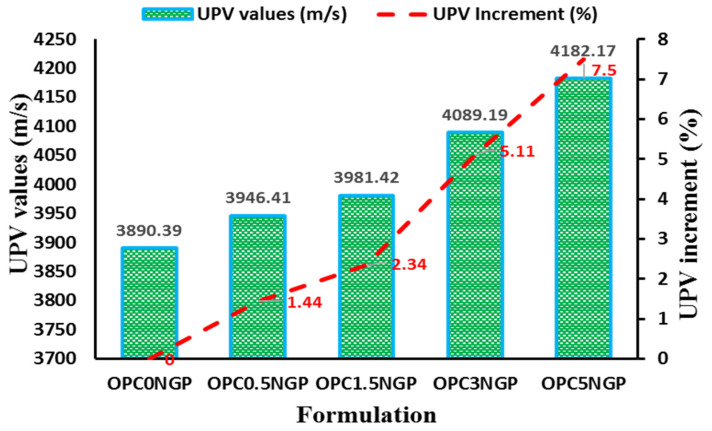
Influence of NGPs on the UPV values.

The UPV is a durability test used to determine the uniformity, quality, and consistency of concrete. It determines a concrete mix’s compactness, homogeneity, and imperfections, such as voids and cracks. The concrete homogeneity was examined from whether the pulse wave passes at a higher or lower velocity through the specimen [53,58]. When the UPV value lies in the range of “3660–4575 m/s”, the concrete specimen is said to be of good quality [59]. Figure 11 shows the quality of the NGP-modified concrete containing different dosages of NGPs compared to the control mix. The results show that the UPV value enhanced with an increase in the percentage of NGP intrusion. It was found that the percentage increase in UPV values was maximum for the mix prepared containing 5% NGP incorporation, which was reported to be 7.5%, and the minimum increase was noted to be 1.44% of the mix prepared with 0.5% NGP, with reference to the control mix. It was revealed that the velocities of all of the concrete mixes at the 28-day curing period were of good quality. Ahmad, F. [5] incorporated different dosages of NGPs in cementitious composites and reported that the UPV value increased linearly with an increase in the dosages of NGPs. It was revealed that when dosages of NGPs increased from 0% to 5%, the UPV value increased by about 2.5%. Similarly, Devi, S. and R. Khan [53] incorporated GO in cementitious composites and found that the UPV value increased with the increase in GO concentration. Different dosages of GO (i.e., ranging from 0 to 0.08% by weight of cement) was incorporated and the maximum UPV value was obtained was at 0.08% of GO. This increase in the UPV values is owing to the nanofiller effect of NGPs, which reduces the porosity and densifies the concrete composites at the nano level [31,53,60]. Hence, increasing the homogeneity and quality of concrete reflects on the mechanical and durability properties of concrete.

#### 4.4.3. Water Absorption

**Figure 12:**
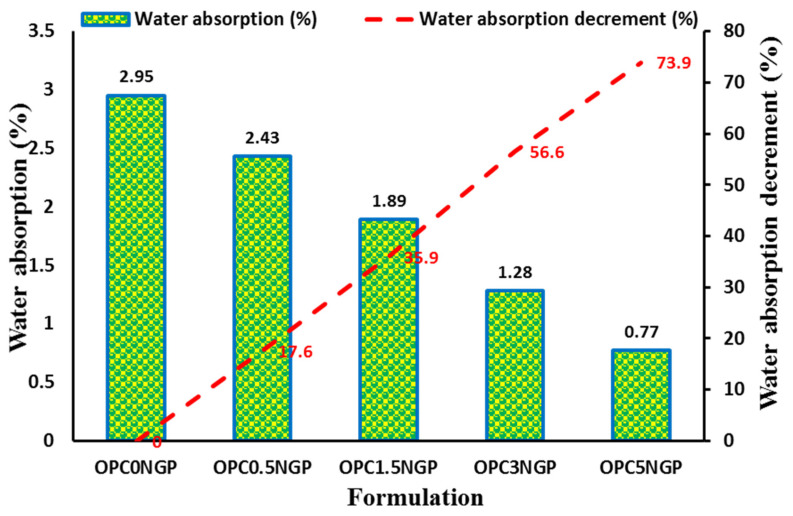
Water absorption (%) of NGP-intruded concrete and percentage decrement in water absorption.

Water absorption is a durability test conducted to measure the amount of water absorbed by cementitious composites. This absorbed water adversely affects the durability behavior of the resulting cementitious materials. Water acts as a medium for the ingression of aggressive agents, such as chloride, sulfate, etc., by capillary actions. In this study, different dosages of NGPs were added to the concrete composites, which showed a substantial decrement in water absorption, as shown in Figure 12. NGP-modified concrete has less water absorption with reference to the control mix [61,62].

It was observed that the control sample (OPC0NGP) without NGP intrusion indicated a maximum water absorption by an amount of 2.95% at the 28-day curing period. OPC5NGP showed the lowest water absorption of 0.77%, whereas OPC0.5NGP revealed the maximum water absorption of 2.43% compared to the control sample without nano intrusion. Percentage reductions in water absorption of NGP-intruded cementitious composites were reported to be 17.62%, 35.93%, 56.61%, and 73.89% for OPC0.5NGP, OPC1.5NGP, OPC3NGP, and OPC5NGP, respectively, compared to the control mix (OPC0NGP) without NGP intrusion. The influence of NGPs upon the water absorption of concrete is consistent with the findings of previously published studies [20,54,61,62]. Devi, S. and R. Khan [53] examined the water absorption and permeability of the nano-reinforced concrete composites containing different dosages of graphene oxide (GO) and reported that the reduction in water absorption was recorded with increasing content of GO in comparison with the control mix. This decrease in water absorption was attributed to the pore-refining effect of GO. NGPs significantly improve the resistance against water absorption by reinforcing the concrete composites at the nano level, reducing the porosity, due to the filler effect, and accelerating the growth of hydration products, owing to the available nucleation site, and densifying the concrete matrix.

#### 4.4.4. External Sulfate Attack

**Figure 13:**
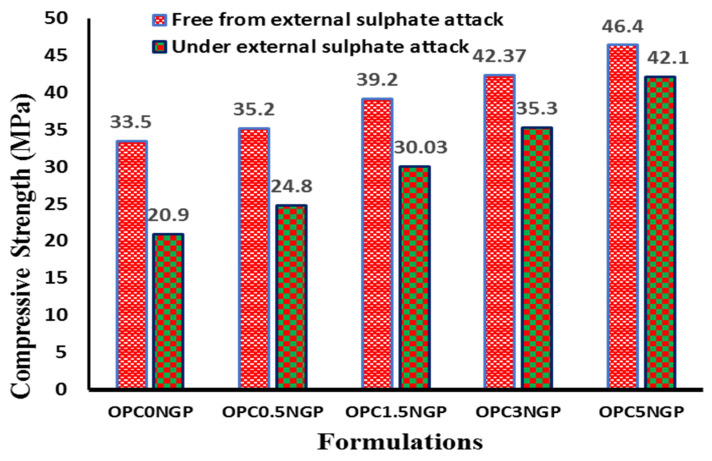
Influence of NGPs on compressive strength of concrete mixes submersed in sodium sulfate solution for 28 days.

An external sulfate attack test was performed to check the resistance of concrete against sulfate. A sulfate attack adversely affects the durability properties of concrete. Sulfate from external sources, such as soil, seawater, underground water, or swamp water reacts with hydration compounds, i.e., calcium aluminate and calcium hydroxide hydrates, and, as a result, expansive delayed ettringite is formed. Expansive ettringite formation causes an increase in solid volume, which results in cracking, expansion, mass loss, and disintegration of concrete [63,64]. The ettringite formation significantly deteriorates the microstructure of cementitious composites. The ettringite growth exerts stresses on the nearby matrix, causing an increase in pores and loss of strength of cementitious composites. Resistance of concrete against a sulfate attack mainly depends on water absorption capacity and permeability [61]. Figure 13 reported the strength reduction of nano-reinforced concrete caused by an external sulfate attack.

A maximum decrease in compressive strength of about 37.43% was reported in the control formulation, without nano intrusions. In control formulation (OPC0NGP), compressive strength of 28-day cured concrete reduced from 33.5 to 20.96 MPa after submersion of the specimen for 28 days in a sodium sulfate solution. This decrement in strength is due to the susceptibility of the control specimens to admittance of sulfate ions from the solution, and no reinforcement is there at the nano level that can obstruct the stresses employed by expansive ettringite; hence, resulting in loss of high strength. The ingression of sulfate ions into the control concrete specimen can be attributed to its least resistance against waster absorptivity (Figure 12).

OPC5NGP showed maximum resistance against an external sulfate attack, and only a 9.22% reduction in strength loss was observed. In other mixes, such as OPC0.5NGP, OPC1.5NGP, and OPC3NGP, approximately 29.4%, 23.38%, and 16.57% strength reductions were reported, respectively. It was observed that concrete samples containing different dosages of NGPs have less percentage reductions in compressive strength, with reference to the control sample without nano intrusion. This decrement in strength reduction is owed to the presence of NGPs. The existence of NGPs decreases water absorption, as shown in Figure 12, consequently reducing the ingression of sulfate ions. Akbar, A., K. Liew, F. Farooq, and R. A. Khushnood [20] studied the effect of using a hybrid of GNMPs and MWCNTs on the behavior of concrete subjected to an external sulfate attack. It was found that the compressive strength of cementitious composites without GNMPs was reduced to 36.3% when exposed to a sulfate attack; however, this loss in compressive strength decreased to 12.2% when GNMPs were incorporated. This decrement in compressive strength loss was attributed to the pore refining and filler effect, and barrier properties of nanoparticles, which strengthened the concrete composites. NGPs, due to their filler effects, provide effective strengthening at the nano level, and increase the density and reduce the porosity of composites; therefore, providing excellent resistance against ingression of a sodium sulfate solution and resistance against deterioration of concrete composites [20].

### 4.5. Microstructure Investigation Using SEM–EDX Analysis

**Figure 14:**
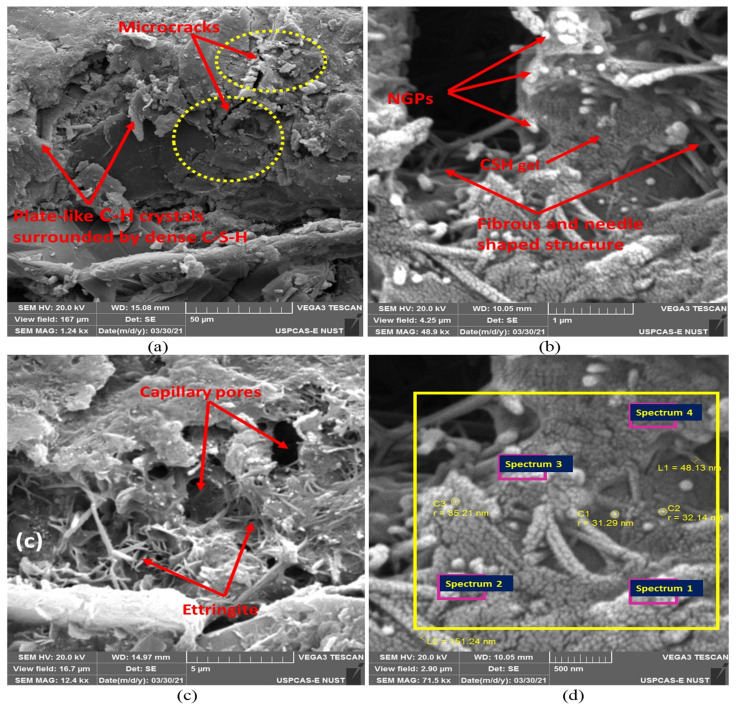
(**a**–**d**) SEM micrographs of NGP-reinforced composites containing 5% NGPs. Reproduced with permission from Ref. [5]. Copyright (2021), Elsevier Ltd.

**Figure 15:**
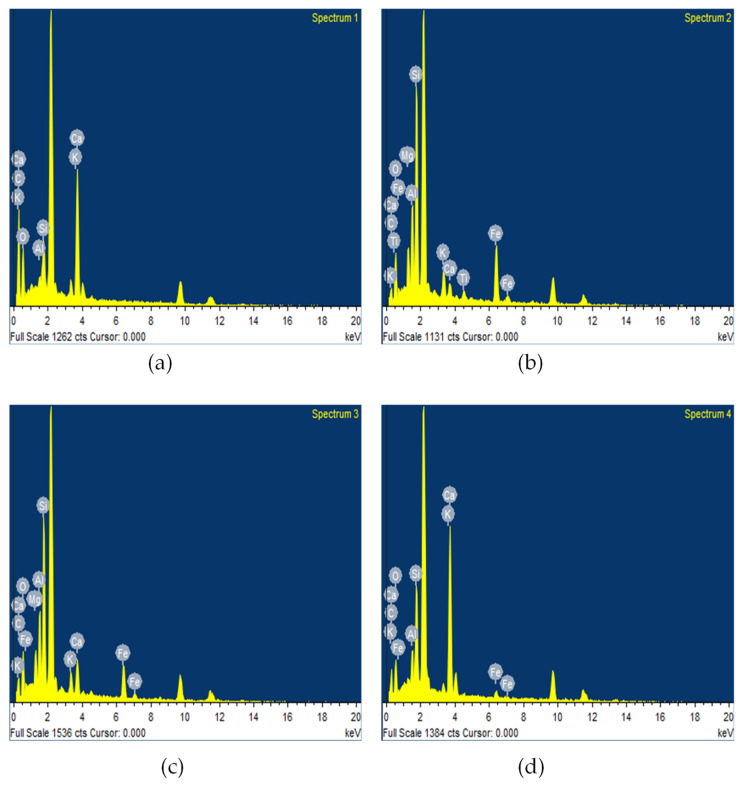
(**a**–**d**) EDX spectra of the SEM micrograph indicated in Figure 14d. Reproduced with permission from Ref. [5]. Copyright (2021), Elsevier Ltd.

Microstructural analysis were carried out to analyze the effects of NGPs at the nano level. In this study, a microstructural investigation was carried for mix containing 5% NGPs because of its extensive performance as a reinforcing agent, acting as a pore refining and densifying agent at the nano level. The SEM and EDX analysis were carried out in the “Material characterization lab-USPCAS-E NUST, Pakistan”. These are the general features of the SEM device used: VEGA3 TESKAN, resolving power (max): 2.3 nm, energy: 30 KV, and magnification (max): 300,000×); whereas the EDX device: EDX with SEM (JSM910) INSA200/Oxford instruments, U.K, and analysis range Boron to Uranium. The samples were examined via several magnification ranges, i.e., 50 μm, 5 μm, 1 μm, and 500 nm near ITZ. The SEM micrographs of specimens containing 5% NGPs are shown in Figure 14. In Figure 14a, dense platy hydrates were surrounded by dense C-S-H gel combined with microcracks. However, the microstructure of the mix containing 5% NGPs displays substantial improvement with densified hydrated crystals and lessor pores, which led to C-S-H formation in combination with CH platy crystals and ettringite (fibrous and needle shape crystals) in Figure 14b–d, respectively. The EDX spectra (i.e., from spectrum 1 to 4) of the SEM micrograph are shown in Figure 14d, whereas Table 8 shows the corresponding elemental composition of each spectra. The square EDX spectra are shown in Figure 15a–d. The high amount of carbon quantity for each spectrum indicates the presence of NGPs, as shown in Table 8. The influence of NGPs on the performance of concrete is consistent with the findings of past studies [4,14,15,65]. Ahmad, F. [5] found that with the varying dosages of NGPs (i.e., 0% to 5%) in concrete composites, the hydrated crystals continued to become thicker, complex, and stacking one over the other, resulting in reinforcement of concrete composite. Petrounias, P., P. P. Giannakopoulou, A. Rogkala, P. Lampropoulou, B. Tsikouras, I. Rigopoulos, and K. Hatzipanagiotou [66] conducted a microstructural investigation to analyze the effects of using various types of recycled materials on the mechanical and petrographic properties of the resultant concrete. It was reported that using beer green glass particles in cementitious composites produces lower cohesion/a weak bond between the cement paste and beer green glass particles compared to glass with quartz primer. Cui, X., S. Sun, B. Han, X. Yu, J. Ouyang, S. Zeng, and J. Ou [15] studied the effects of NGPs on concrete composites and reported that, with the increasing percentages of NGPs, the cement matrix continued to become denser. Figure 14 shows that by incorporating NGPs in the cementitious composites, the hydrated crystals become massive, thicker, stacking over each other, and complex, inferring that NGPs are promising reinforcement agents in concrete.

## 5. Conclusions

This study examined the effects of NGPs on the fresh and hardened properties of concrete, including workability, air content, hardened density, compressive strength, split tensile strength, and flexural strength. Furthermore, durability properties of nano-reinforced concrete, such as sorptivity coefficient, UPV, water absorption, and external sulfate attacks, were explored. Based on the findings, the following conclusions can be deducted.

- The workability of NGP-modified concrete decreased as compared to the control sample due to the fine particle size and large surface area of NGPs. The decreasing trend in workability was found with increasing NGP dosages. Moreover, a decrement in percentage air content was observed with increasing dosages of NGPs. The concrete mix with 5% NGPs indicated the maximum reduction in air content, about 33.83%, with reference to the control mix.
- The hardened density of concrete increased with the rise in dosages of NGPs. The maximum increase in density of about 11.46% was observed for the sample containing 5% NGPs, with reference to the control mix. The compressive and split tensile strength of the concrete also increased. Compressive and tensile strength values increased in the range of 5–38.5% and 8.25–31.6%, respectively. This is because of the intrusion of NGPs, which strengthened the concrete composites at the nano level; increasing the density and hardness of concrete make NGP a promising reinforcing agent in cementitious composites. In addition, the mix containing 5% NGP intrusion showed a maximum enhancement in flexural strength of 44.34%.
- The incorporation of NGPs significantly reduced the sorptivity value (32.3% reduction for 5% NGPs intrusion) due to its filler effect. The values of UPV also improved. The maximum increase in the UPV value was from 3890 m/s to 4182 m/s at 5% intrusion of NGPs.
- The addition of NGPs in concrete composites proved effective at reducing water absorption. The mix with 5% NGPs showed a 73.9% reduction in water absorption compared to the control mix.
- The current study focused on the physical and mechanical characteristics of NGP-incorporated concrete. The NGPs can potentially be used to achieve high-density concrete with improved performance for sustainability and nuclear infrastructure. It is strongly recommended to see the influence of NGPs in the freeze–thaw cycles, alkali–silica reaction, salt scaling, and carbonation; the optimum dosage levels of NGPs for future implementations; the effects of NGPs on drying shrinkage of cementitious composites; the influence of different water to cement (W/C) ratios on NGP-modified concrete composites; NGP-modified concrete at low and high W/C ratios; statistical analyses of NGP-incorporated concrete specimens to analyze the deviation from the experimental results.
- The paradigm shift in regression models using machine learning significantly contributes to solving engineering problems [67,68,69,70,71]. The current study investigated the effect of changing dosages of NGPs on the mechanical characteristics of concrete. To avoid the laborious testing, the data used in the manuscript, alongside other similar data from experiments or the literature, can potentially be used to develop multiple artificial intelligent models, according to previous literature [31,71,72,73,74,75,76,77].

## Figures and Tables

**Table 1:** Physical and chemical properties of OPC.

Chemical Composition	Content	Physical Properties	Results
(Oxides)	(%)		
SiO_2_	20.6	Specific surface area (m^2^/kg)	321
CaO	65.58	Specific gravity	3.14
Fe_2_O_3_	3.1	Initial setting time (min)	185
Al_2_O_3_	5.1	Final setting time (min)	241
MgO	2.42	Consistency (%)	29.15
SO_3_	1.64	Soundness (%)	0.103
K_2_O	0.72	Fineness modulus (%)	93.3
Na_2_O	0.23	Compressive strength (28 days MPa)	46.56
Loss on ignition (LOI)	0.61	-	-

**Table 2:** Physical characteristics of aggregates.

Property	Coarse Aggregate	Fine Aggregate
Min. nominal size (mm)	4.74	4.72
Max. nominal size (mm)	20	0.074
SSD water absorption (%)	1.08	0.5
Specific gravity	2.71	2.78
Shape	Angular	_
Color	Dark	Dark
Bulk density (lb/ft^3^)	94.05	100
Fineness modulus	NIL	2.27
Aggregate impact value (%)	25.43	NIL
Aggregate crushing value (%)	27.42	NIL

**Table 3:** Elemental composition of AG.

Elements	Weight (%)	Atomic (%)
C	67.61	74.8
O	32.11	24.57
K	0.34	0.14
Na	0.20	0.09
Ca	0.90	0.12
Mo	0.84	0.28
Total	100	100

**Table 4:** Dispersion scheme mix ratios.

Sample Name	NGPs:AG
R1	1:0
R2	1:0.2
R3	1:0.4
R4	1:0.6
R5	1:0.8
R6	1:1

**Table 5:** Concrete mix proportions in Kg/m^3^.

Formulation	Cement	NGPs	Fine Aggregate	Coarse Aggregate	Water	Superplasticizer (mL)
OPC0NGP	384	0	715	1113	173	192
OPC0.5NGP	384	1.92	715	1113	173	192
OPC1.5NGP	384	5.76	715	1113	173	192
OPC3NGP	384	11.52	715	1113	173	192
OPC5NGP	384	19.2	715	1113	173	192

**Table 6:** Chemical composition of NGPs.

Elements	Nano Graphite Platelets (NGPs)	
	Atomic (%)	Weight (%)
C	91.35	85.83
Si	1.37	2.98
Ca	0.59	1.70
Al	0.31	0.74
O	5.45	5.76
Fe	0.56	2.04
Mg	0.23	0.62
S	0.14	0.32
Total	100	100

**Table 7:** Mineral oxide composition of NGPs.

Oxides	CaO	SiO_2_	Fe_2_O_3_	K_2_O	MoO_3_	CuO	TiO_2_	ZnO	ZrO_2_	MnO
Weight (%)	25.52	36.4	32.67	2.177	0.244	0.612	1.434	0.105	0.125	0.165

**Table 8:** Elemental entities obtained from SEM–EDX spectroscopy (S; spectrum).

Elements	S1	S2	S3	S4
C K	50.71	34.39	34.51	31.92
O K	34.42	29.64	33.11	36.48
Al K	0.68	4.43	5.51	2.68
Si K	1.99	15.35	12.47	6.33
K	1.22	2.86	2.36	0.99
Ca K	10.98	1.8	4.4	19.26
Mg K	_	3.64	3.11	_
Fe K	_	6.52	4.54	2.35
Ti K	_	1.37	_	_
Total	100	100	100	100

## Abbreviations

NGPs	nano graphite platelets
GNPs	graphite nano platelets
GO	graphene oxide
AG	acacia gum
GONPs	graphene oxide nano platelets
CNTs	carbon nanotubes
CNFs	carbon nanofibers
GNMPs	graphite nano-micro particles
OPC	Ordinary Portland Cement
CH	calcium hydroxide
UPV	ultrasonic pulse velocity
UHPC	ultrahigh performance concrete
ITZ	interfacial transition zone
EDX	energy dispersive x-rays
XRD	X-ray diffraction
XRF	X-ray fluorescence
MWCNTs	multi-walled carbon nanotubes
UV–Vis	ultra violet visible spectroscopy
SEM	scanning electron microscopy
C-S-H	calcium silicate hydrate
